# Warming trends and shortened growing seasons: integrating four decades of observations and model simulations to develop wheat adaptation strategies in semi-arid Pakistan

**DOI:** 10.1038/s41598-026-36853-z

**Published:** 2026-02-04

**Authors:** Mukhtar Ahmed, Aashir Sameen, Ahmed M.S. Kheir

**Affiliations:** 1 Department of Agronomy, Pir Mehr Ali Shah Arid Agriculture University, Rawalpindi, 46300 Pakistan; 2 Swedish University of Agricultural Sciences Umea, Umea, Sweden; 3 Julius Kühn Institute (JKI)—Federal Research Centre for Cultivated Plants, Institute for Strategies and Technology Assessment, 14532 Kleinmachnow, Germany; 4 Soils, Water and Environment Research Institute, Agricultural Research Center, Giza, 12112 Egypt

**Keywords:** Wheat, Climate change, Growing degree days, Crop phenology, Grain yield, Sowing date adaptation, Climate sciences, Ecology, Ecology, Environmental sciences, Plant sciences

## Abstract

Climate change is increasingly affecting wheat phenology and productivity in semi-arid regions. This study assessed the impact of long-term climate warming on wheat developmental stages, yield, and management options in rainfed systems of Pakistan. Fourty one years (1980–2020) of phenological (days to anthesis and maturity), yield, and meteorological data (minimum and maximum temperature, and rainfall) were analyzed for two locations: Islamabad (33.67° N, 73.12° E) and Chakwal (32.93° N, 72.86° E). Growing degree days (GDD) were calculated using cardinal temperatures of 4 °C (base), 13.2 °C (optimum), and 35 °C (ceiling). Cumulative GDD ranged from 1,679 to 2,637 °C-days at Chakwal and 2,000–2,500 °C-days at Islamabad. Increasing mean temperatures (18.0 °C at Chakwal and 17.8 °C at Islamabad) were associated with accelerated crop development, reducing days to anthesis (from 127 to 70 days at Chakwal and 133 to 74 days at Islamabad) and maturity (from 155 to 85 days at Chakwal and 163 to 93 days at Islamabad). Consequently, grain yield declined from 2.0 to 1.5 t ha⁻¹ at Chakwal and from 2.3 to 1.4 t ha⁻¹ at Islamabad. Simulated sowing date scenarios showed that early sowing avoided heat stress, whereas delayed sowing caused yield losses of up to 27% at Islamabad and 25% at Chakwal. The results indicate that advancing sowing to 1–15 October and adopting climate-resilient wheat cultivars informed by process-based models can help mitigate warming-induced yield losses.

**Supplementary Information:**

The online version contains supplementary material available at 10.1038/s41598-026-36853-z.

## Introduction

The Sixth Assessment Report (AR6) of the Intergovernmental Panel on Climate Change (IPCC) indicates that global mean temperature is likely to exceed 1.5 °C above pre-industrial levels by the early 2030 s, highlighting the urgency of substantial and sustained reductions in greenhouse gas (GHG) emissions. The AR6 further identifies 2010–2019 as a decade of historic climate extremes, including record heatwaves, disrupted rainfall patterns, and increased frequency and intensity of extreme weather events such as floods. During this period, global GHG emissions, particularly carbon dioxide (CO₂), reached unprecedented levels, primarily due to anthropogenic activities such as fossil fuel combustion and land-use changes^1–9^. Changes in temperature regimes and precipitation patterns have altered crop phenology, shortened growing seasons, and increased environmental stressors, particularly in South Asia^10^. Temperature fluctuations have also been linked to shifts in soil biodiversity and reductions in wheat yield, with a 1 °C increase in seasonal average temperature associated with an approximate 6% yield decline^9,11^.

Agriculture is highly vulnerable to climate change because crop growth and productivity are directly influenced by temperature and water availability. Wheat (*Triticum aestivum* L.), a major rabi-season crop in Pakistan, is typically sown from October to December and harvested from April to May. It is cultivated on approximately 8.9 million hectares, contributing 7.8% to the agricultural value added and 1.8% to the national GDP. In rainfed regions, wheat is sown at the start of winter to fulfil its chilling requirement. In Punjab province, wheat was grown on 5,884 thousand hectares under irrigated and 678 thousand hectares under non-irrigated conditions during 2021–22, with Rawalpindi division accounting for 40 hectares (irrigated) and 16,263 hectares (non-irrigated). Wheat provides over 20% of calories and protein in human diets^12–15^.

Rising temperatures and delayed or traditional sowing expose wheat to heat and drought stress, particularly during crop establishment and the anthesis-to-grain-filling period, resulting in significant yield reductions^16–18^. Wheat is highly sensitive to temperature, with optimal growth occurring between 12 and 22 °C during anthesis and grain filling. Exposure to temperatures above 30 °C during these stages can induce premature senescence and reduce grain weight. Accumulated heat units, or growing degree days (GDD), calculated by summing daily average temperatures above a base of 4 °C, are widely used to assess the impact of temperature on crop phenology and yield. GDD also provides guidance for optimal sowing and harvest dates under changing climatic conditions^19–24^. Heat stress in wheat can be mitigated by adopting early-maturing cultivars or adjusting sowing dates. Pre- and post-anthesis exposure to temperatures above 30 °C reduces the grain-filling rate, thereby lowering final yield. Modeling studies in Punjab have shown that late-sown wheat is particularly vulnerable to heat stress, whereas early-maturing cultivars and adjusted sowing dates can help minimize yield losses^25–29^.

Investigating long-term impacts of climate change on wheat phenology and yield, particularly in rainfed systems from 1980 to 2020, provides valuable insights into cumulative climate effects that short-term studies may overlook. Such analyses can inform adaptation strategies, including optimized sowing dates, selection of climate-resilient cultivars, and carbon management practices^9,30–37^. Regional-scale monitoring and yield forecasting are essential for policy-making, commercial planning, and ensuring food security. Recent studies highlight the importance of advanced modeling techniques, such as deep learning and Bayesian ensemble approaches, for accurate yield prediction and decision support^38^. Wang et al^39^. reported importance of timely and accurate forecasting of crop yields which is crucial to food security and sustainable development in the agricultural sector. Thus, they suggested use of deep learning model in the accurate prediction of winter wheat yield. Similarly, Huang et al^40^. indicated the importance of Bayesian posterior-based ensemble Kalman filter (EnKF) in the crop yield estimation.

Despite extensive research on climate impacts on wheat globally, few studies have quantified long-term climate effects in the rainfed regions of Pakistan. This study aims to address this gap with four objectives: (i) quantify decadal changes in wheat growing season temperature; (ii) examine relationships between long-term temperature trends and wheat phenology and yield; (iii) quantify variability in heat units (GDD) and their relationship with phenology and grain yield; and (iv) optimize sowing dates based on long-term data. Historical weather, wheat growth, and yield records were analyzed to achieve these objectives, using regression and simulation approaches to identify trends and propose climate-adaptive strategies for wheat production in semi-arid Pakistan.

## Materials and methods

### Study sites description

**Fig. 1:**
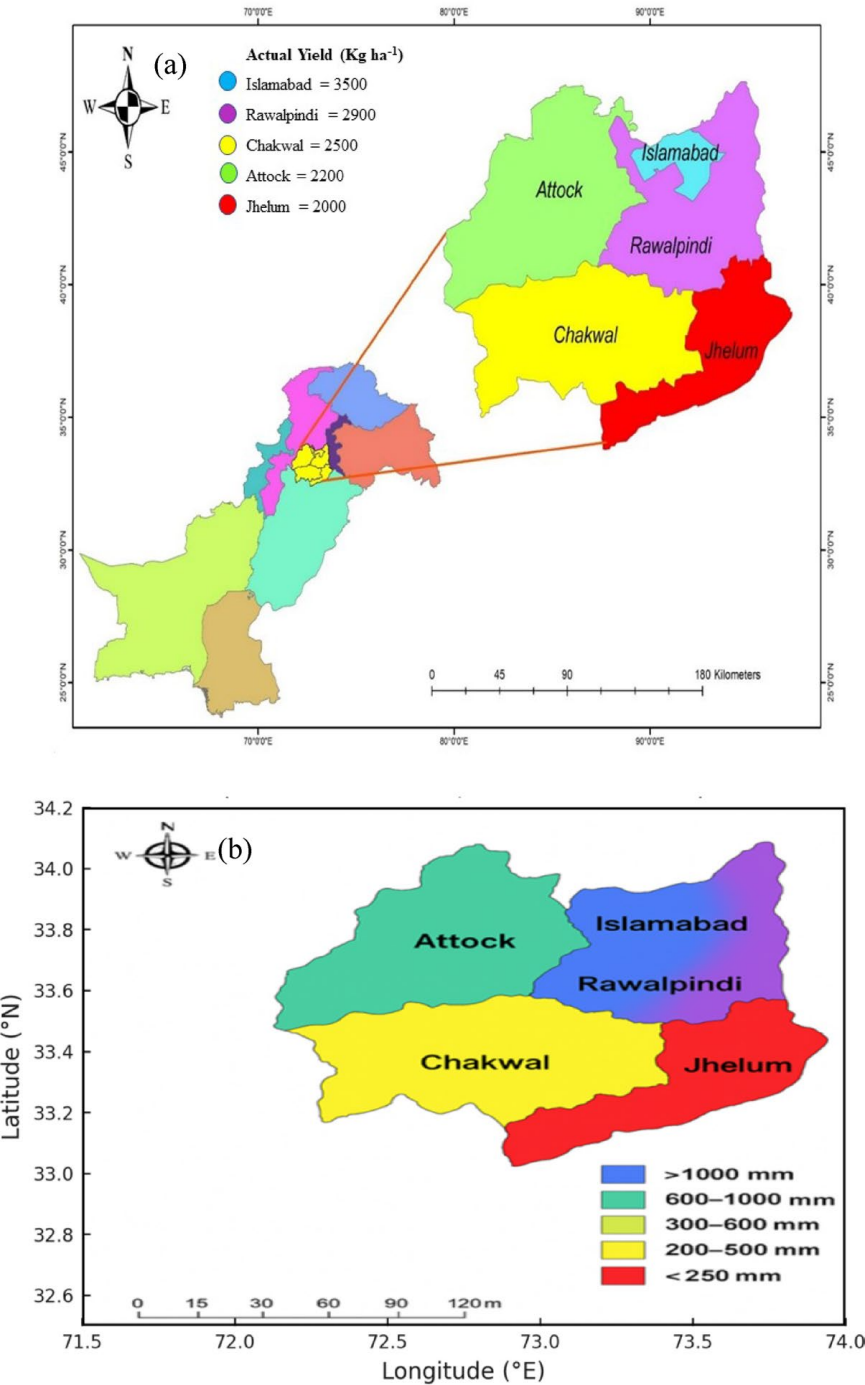
Study area showing (a) district-wise actual wheat yield and (b) spatial distribution of mean annual rainfall. Maps were generated using ArcGIS Desktop software (version 10.8; https://www.esri.com/arcgis).

The study was conducted at two rainfed locations in Pakistan: Islamabad (33.67° N, 73.13° E) and Chakwal (32.93° N, 72.86° E). Both sites are located within the semi-arid Pothwar Plateau and receive mean annual rainfall ranging from approximately 250 to 750 mm (Fig. 1).

Soils at Islamabad are loamy, while those at Chakwal are sandy clay loam. According to the USDA soil taxonomy, Islamabad soils are classified as Entisols (Ustorthents), whereas Chakwal soils are Alfisols (Haplustalfs). Soil physicochemical properties are presented in Table 1. Descriptive statistics of climatic variables, growing degree days (GDD; °C day), wheat phenology (days to anthesis and maturity), and grain yield for the period 1980–2020 are shown in Tables 2 and 3.

**Table 1:** Soil physical and hydraulic properties at two depths at Islamabad and Chakwal.

Parameter	Unit	Islamabad (0–30 cm)	Islamabad (30–60 cm)	Chakwal (0–30 cm)	Chakwal (30–60 cm)
Organic carbon	%	0.89	0.62	0.69	0.46
Silt	%	33	33	22	20
Sand	%	35	35	56	56
Clay	%	32	32	22	24
Texture class	—	Loam	Loam	Sandy clay loam	Sandy clay loam
Bulk density	g cm⁻³	1.33	1.49	1.37	1.6
SLL† (lower limit)	cm³ cm⁻³	0.12	0.13	0.07	0.08
SDUL‡ (drainage upper limit)	cm³ cm⁻³	0.29	0.26	0.22	0.19
Saturated water content	cm³ cm⁻³	0.44	0.37	0.43	0.33

Note:† SLL = Soil lower limit (wilting point).

‡ SDUL = Soil drainage upper limit (field capacity).

**Table 2:** Descriptive statistics of Climatic variables, growing degree days (^o^C day^− 1^) phenology (days to anthesis and days to maturity) and grain yield of wheat during the period of 1980–2020 at Islamabad.

Parameters	Range	Minimum	Maximum	Mean	Std. Deviation	Variance	Skewness	Kurtosis
Average T (^o^C)	7.0	16.5	23.5	19.3	2.17	4.70	0.60	−1.03
Maximum T (^o^C)	5.9	22.7	28.6	25.4	1.33	1.76	0.17	−0.32
Minimum T (^o^C)	3.6	8.8	12.4	10.3	0.89	0.79	0.61	−0.20
Average temp 7 before Anthesis (^o^C)	9.9	16.4	26.3	22.2	2.44	5.98	−0.42	−0.55
Average temp 7 after Anthesis (^o^C)	13.3	16.3	29.6	23.3	2.62	6.87	−0.26	0.16
Average temp 7 before Maturity (^o^C)	11.6	14.2	25.9	19.6	2.51	6.31	0.20	−0.04
Average temp 7 after Maturity (^o^C)	7.7	24.1	31.9	28.5	1.83	3.34	−0.47	0.11
Rainfall (mm)	1855.9	96.1	1952.0	427.9	311.84	97247.16	3.16	13.88
Days to Maturity	70.0	93.0	163.0	135.0	18.10	327.72	−0.81	−0.18
Days to Anthesis	70.0	63.0	133.0	105.0	18.10	327.72	−0.81	−0.18
Grain Yield (t ha^− 1^)	1.49	1.48	2.97	2.25	0.46	0.21	−0.09	−1.39
Growing Degree Days (^o^C Day^− 1^)	440	2023	2463	2252	120	14,344	0.01	−1.06

**Table 3:** Descriptive statistics of Climatic variables, growing degree days (^o^C day^− 1^) phenology (days to anthesis and days to maturity) and grain yield of wheat during the period of 1980–2020 at Chakwal.

Parameters	Range	Minimum	Maximum	Mean	Std. Deviation	Variance	Skewness	Kurtosis
Average T (^o^C)	7.4	14.5	21.9	18.3	1.55	2.40	−0.14	0.252
Maximum T (^o^C)	11.3	18.5	29.8	25.4	2.45	6.03	−0.62	0.678
Minimum T (^o^C)	5.8	8.3	14.1	11.1	1.25	1.58	−0.09	0.005
Average temp 7 before Anthesis (^o^C)	11.0	16.6	27.6	21.1	2.97	8.87	0.74	−0.205
Average temp 7 after Anthesis (^o^C)	14.3	16.6	30.9	23.0	3.37	11.38	0.47	−0.372
Average temp 7 before Maturity (^o^C)	11.1	20.0	31.1	26.5	3.03	9.22	−0.31	−0.589
Average temp 7 after Maturity (^o^C)	14.7	19.7	34.4	27.8	3.33	11.10	−0.35	0.243
Rainfall (mm)	1378.9	71.0	1449.9	296.6	227.43	51727.5	3.48	16.391
Days to Maturity	67	60	127	100.1	17.30	299.5	−0.79	−0.360
Days to Anthesis	70	85	155	127.2	17.82	317.8	−0.76	−0.315
Grain Yield (t ha^− 1^)	1.04	1.04	2.08	1.57	0.32	0.10	−0.09	−1.387
Growing Degree Days	958	1679	2637	2214.5	211.83	44871.9	−0.72	0.367

### Wheat phenology and yield data

Long-term wheat phenology and yield data (1980–2020) were compiled from multiple sources, including the Economic Survey of Pakistan^41^,, FAOSTAT^42^, the Agriculture Marketing Information System of Pakistan, (http://www.amis.pk/Agristatistics/Statistics.aspx), and published literature and theses retrieved from ScienceDirect, Springer, and the HEC repository (http://prr.hec.gov.pk/jspui/handle/123456789/9). These datasets were cross-checked to ensure consistency and completeness.

### Meteorological data

Daily maximum (Tmax) and minimum (Tmin) temperature data for 1980–2020 were obtained from the Pakistan Meteorological Department. Data were analyzed for the wheat growing season (15 October–30 April). Mean temperatures before anthesis and before maturity were calculated to assess terminal heat stress. Long-term baseline values for days to anthesis (DTA), days to maturity (DTM), seasonal temperature, and rainfall were computed by averaging the entire dataset for each location.

### Growing degree days (GDD)

Growing degree days were calculated using the cardinal temperature approach to quantify heat accumulation during wheat development. Cardinal temperatures proposed by Porter and Gawith^43^ for wheat were adopted, with stage-specific values applied for vegetative, reproductive, and grain-filling phases. These includes, T_base_ = 4 °C, T_optimum_ = 22.0 °C and T_ceiling_ = 32.7 °C for the phenological phases from sowing to the start of anthesis. From anthesis to grain filling we used ceiling temperature of T_base_ = 9.5 °C, T_optimum_ = 21.0 °C and T_ceiling_ = 31.0 °C. However, from grain filling to maturity GDD was calculated by considering ceiling temperature of T_base_ = 9.2 °C, T_optimum_ =20.7 °C and T_ceiling_ = 35.4 °C. GDD was calculated from sowing (15 October) to maturity (30 April), corresponding to a total growing period of 196 days. Days with temperatures exceeding the ceiling threshold (35 °C during grain filling) were assigned a GDD value of zero. GDD was calculated using following Eq. (1):

1 $$\:\mathrm{GDD\:=}\frac{\:\left({\mathrm{T}}_{\mathrm{max}}\mathrm{+}{\mathrm{T}}_{\mathrm{min}}\right)}{\mathrm{2}}\mathrm{-}{\mathrm{T}}_{\mathrm{base}}\:$$

Where,

T_max_ = Daily maximum Temperature (^o^C).

T_min_ = Daily minimum Temperature (^o^C).

T_b_ = Base Temperature (4 °C).

### Sowing dates scenarios

Five sowing date (SD) scenarios were evaluated to assess temperature effects on wheat phenology and yield: 15 October (SD1), 25 October (SD2), 4 November (SD3), 14 November (SD4), and 24 November (SD5). Long-term average days to anthesis were fixed at 105 days for Islamabad and 100 days for Chakwal. Average days to maturity were 135 days for Islamabad and 127 days for Chakwal (Table 1). Early, normal, and late sowing categories were defined based on regional practices. To capture varietal diversity over the study period, major rainfed wheat cultivars grown in the region were aggregated and regionally calibrated in the model.

### Crop model simulation

Wheat phenology and grain yield were simulated using the DSSAT v4.8 CSM-CERES-Wheat model. The model integrates weather, soil, crop, and management components to simulate crop growth and yield under varying environmental conditions (https://dssat.net/).

## Model calibration

Model calibration was performed using observed long-term phenology and yield data (1980–2020). Cultivar-specific genetic coefficients related to thermal time, photoperiod sensitivity, and grain filling were adjusted through trial-and-error to minimize discrepancies between simulated and observed values.

## Model validation

The calibrated model was evaluated using independent subsets of observed data. Model performance was assessed using the coefficient of determination (R²), root mean square error (RMSE), mean absolute error (MAE), and bias. Linear regression between simulated and observed values was also conducted. Validation plots were generated using R (ggplot2), with performance statistics annotated. Following formulas were used to calculate R^2^, RMSE, MAE and bias:

2 $$\:{R}^{2}={\left(\frac{{\sum\:}_{i}\left({O}_{i}-\stackrel{-}{O}\right)\left({S}_{i}-\stackrel{-}{S}\right)}{\sqrt{{\sum\:}_{i}{\left({O}_{i}-\stackrel{-}{O}\right)}^{2\:}{{\sum\:}_{i}\left({S}_{i}-\stackrel{-}{S}\right)}^{2}}}\right)}^{2}$$

3 $$\:\mathrm{R}\mathrm{M}\mathrm{S}\mathrm{E}=\sqrt{\frac{1}{\mathrm{n}}\sum\:_{\mathrm{i}=1}^{\mathrm{n}}{\left({\mathrm{S}}_{\mathrm{i}}-{\mathrm{O}}_{\mathrm{i}}\right)}^{2}\:}$$

4 $$\:MAE=\frac{1}{n}\sum\:_{i=1}^{n}\left|{S}_{i}-{O}_{i}\right|\:$$

5 $$\:Bias=\frac{1}{n}\sum\:_{i=1}^{n}\left({S}_{i}-{O}_{i}\right)$$

A linear regression (S = a + bO) was also regressed on both simulated and observed values to obtain slope b and intercept a, which show the performance of the simulations in terms of 1:1 line. R was used to create validation graphs using ggplot2, plots of observed vs. simulated using the line of 1:1 (observed = simulated) and a fitted regression line. Regression equation, R^2^, RMSE, MAE and bias were annotated on each plot to portray the overall model performance graphically.

### Temperature and CO₂ sensitivity analysis

Temperature sensitivity scenarios were generated by increasing daily temperatures by + 1, +2, and + 3 °C relative to the historical baseline, consistent with IPCC AR6 projections under moderate (RCP4.5) and high-emission (RCP8.5) scenarios. CO₂ sensitivity was assessed using a baseline concentration of 410 ppm, with elevated levels of 450 ppm and 550 ppm representing near-future and mid-century conditions. Percentage yield loss divided by degree Celsius (^o^C) rise in temperature was computed as:

6 $$\:Yield\:loss\:\left(\mathrm{\%}\:per\:oC\right)=\left(\frac{{Y}_{ambient}-{Y}_{{+1}^{0}C}}{{Y}_{ambient}}\right)\times\:100\:$$

and Y_ambient_ is the simulated yield of grains at the baseline temperature, and Y_+ 1 °C_ is the simulated yield at a + 1 °C temperature increase. This was calculated with specific temperature increases until an average of the yield reduction rate per °C was obtained of each location being studied.

In the same way, the reaction to high levels of CO_2_ was calculated by comparing the yields which were simulated at different levels of incremental CO_2_ levels using the following expression:

7 $$\:Yield\:change\:\left(\mathrm{\%}\:per\:100ppm\right)=\left(\frac{{Y}_{{CO}_{2}+n}-{Y}_{{CO}_{2}}}{{Y}_{{CO}_{2}}}\right)\times\:100$$

where $\:{Y}_{{CO}_{2}}$ is the yield at the baseline CO_2_ level (350 ppm) and $\:{Y}_{{CO}_{2}+n}$ is the yield at the high CO_2_ levels (e.g. +100 ppm increments). This gave a quantitative measure of the percentage change in yield to 1 °C and 100ppm CO_2_ increase in both Islamabad and Chakwal.

### Statistical analysis

Polynomial regression was applied to see the relationship between the independent variables i.e. years, growing degree days (^o^C Day^− 1^), T_mean_ 7 days before anthesis (^o^C), T_mean_ 7 days after anthesis (^o^C) and seasonal mean temperature with dependent variables i.e. phenology and grain yield. Similarly, regression analysis was conducted to depict relationship between days to anthesis and days to maturity with grain yield. Polynomial regression gives better estimates as it uses higher-degree functions of the independent variable, such as squares and cubes which was used to fit the data. Furthermore, it allows for more complex relationships between variables compared to linear regression. Polynomial regression equation used was:

8 $$\:Y={\beta\:}_{0}+{\beta\:}_{1}{X}_{1}+{{\beta\:}_{2}{X}_{1}}^{2}+{{\beta\:}_{3}{X}_{1}}^{3}+\dots\:+{{\beta\:}_{n}{X}_{1}}^{n}+\epsilon\:$$

Where.

Y = dependent variable (grain yield or phenological stage; unit: t ha⁻¹ or days).

Xi = independent variable(s) (e.g., year, growing degree days, temperature variables).

$\:{\beta\:}_{0}$ = intercept (unit: same as Y).

$\:{\beta\:}_{1}$ = regression coefficients for the ith term (unit: Y/X_i_ⁿ).

*n* = degree of polynomial.

ε = random error term, assumed to be normally distributed with mean 0 and variance σ².

Since variables were continuous, normally distributed thus pearson correlation was calculated to see the association between agro-metrological parameters, wheat phenology and grain yield. Descriptive statistics were applied to see the relationship between crop phenology, yield and climatic variables. Statistical analysis was performed by using Statgraphics 19 and Sigma plot 15.

## Results

### Decadal change in wheat seasonal temperature and rainfall (1980–2020)

**Fig. 2:**
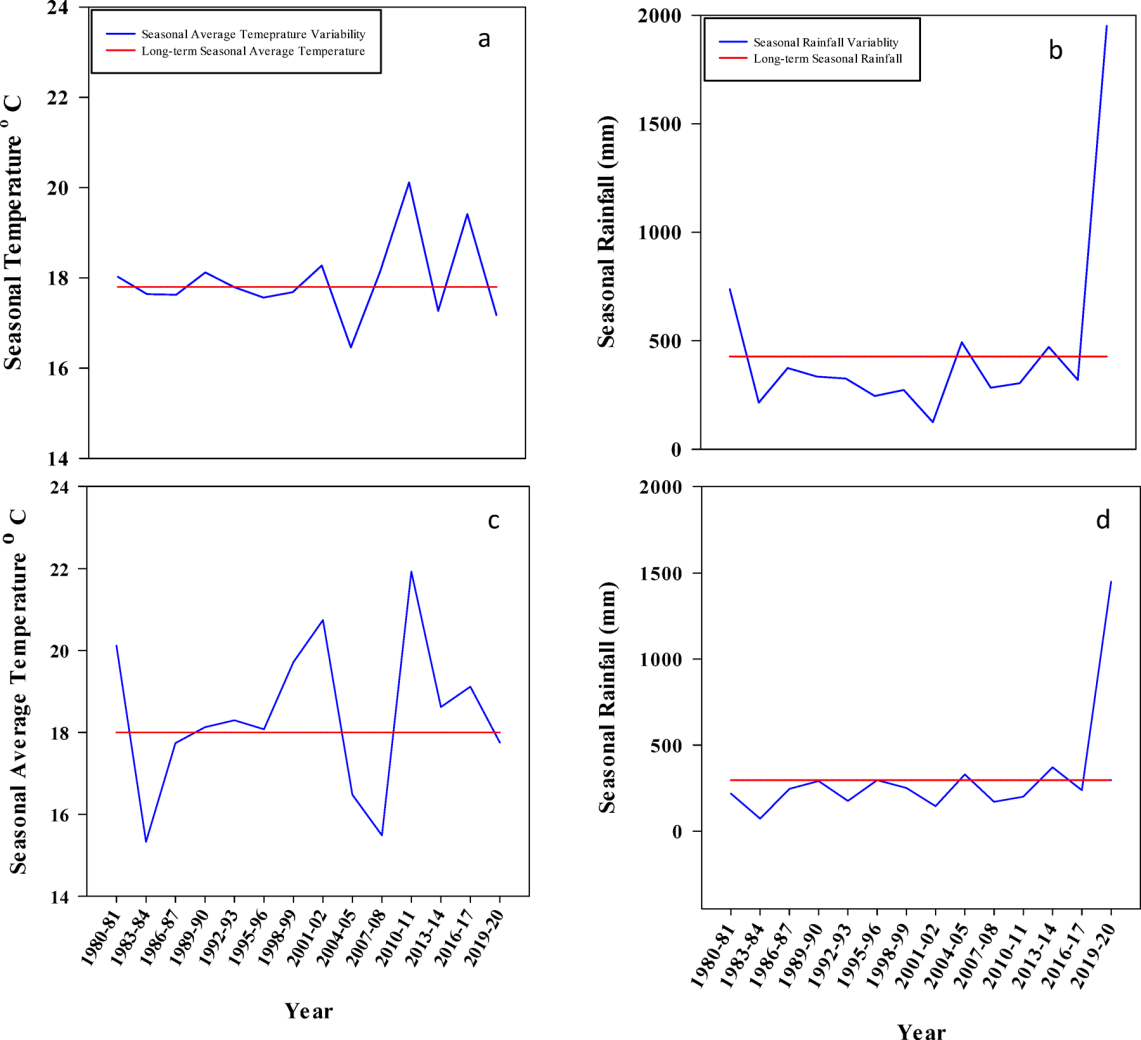
Decadal change in wheat seasonal long-term (1980–2020) average temperature (a and c) and rainfall (mm) (b and d) at Islamabad and Chakwal respectively.

The comparison of mean seasonal temperature during 1980–2020 at the two study locations (Fig. 2a, c) revealed a clear and sustained warming trend with direct implications for wheat production. Mean seasonal temperature showed notable interannual variability around the long-term average; however, a pronounced and consistent increase was observed during the last two decades (2000–2020). The most substantial warming occurred during the spring months (March–May), coinciding with critical wheat growth stages such as flowering and grain filling. During this period, maximum temperatures frequently increased from approximately 26 °C to 37 °C, conditions known to accelerate crop development, shorten the grain-filling duration, and potentially reduce grain yield.

At Islamabad, mean seasonal temperature increased at a rate of approximately 0.40 °C decade⁻¹, resulting in an overall rise of about 1.5 °C over the 41-year study period. Decadal analysis indicated relatively modest warming (~ 0.2 °C) during 1980–1990, followed by a marked acceleration during 2000–2020. In contrast, Chakwal exhibited a more uniform warming pattern, with an increase of approximately 1.0 °C decade⁻¹ throughout the entire study period, indicating persistent thermal stress in this rainfed environment.

Rainfall trends at both locations (Fig. 2b, d) showed an overall decline relative to the long-term mean, although interannual variability was high, with occasional anomalous peaks such as in 2019. The combined occurrence of rising temperatures and declining or erratic rainfall suggests increasing climatic pressure on wheat production in the Pothwar region.

Overall, these results demonstrate a clear warming trend accompanied by increased rainfall variability, with notable site-specific differences between Islamabad and Chakwal. The observed climatic changes are likely to intensify heat stress during sensitive wheat growth stages, emphasizing the need for location-specific adaptation strategies to sustain wheat productivity under changing climate conditions.

### Long-term trends in wheat phenology (days to anthesis and maturity)

**Fig. 3:**
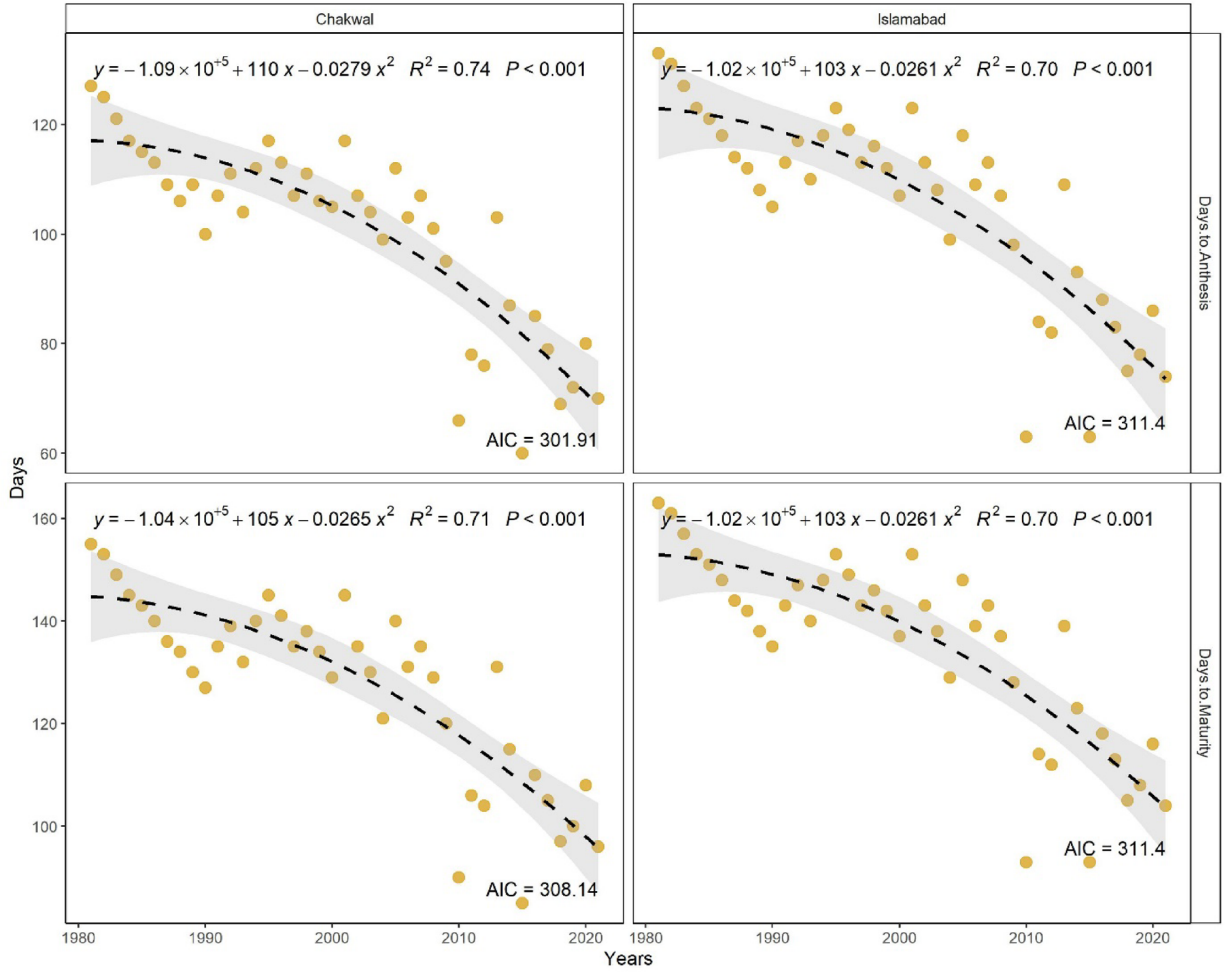
Long-term trend in wheat days to anthesis and days to maturity at Islamabad.

Long-term analysis revealed a significant shortening of the wheat growth period over the last four decades (1980–2020) at both study locations (Fig. 3). Days to anthesis (DTA) showed a strong negative relationship with time, indicating faster crop development under increasing temperatures. At Islamabad, DTA declined from 133 days in 1980–81 to 74 days in 2020–21, with a significant polynomial relationship (R² = 0.70, *p* < 0.01). Similarly, at Chakwal, DTA decreased from 127 to 70 days during the same period (R² = 0.74, *p* < 0.01). Decadal analysis showed that DTA ranged from 133 to 105 days during 1980–1990 and declined to 109–63 days during 2010–2021 at Islamabad, while at Chakwal it decreased from 127 to 100 to 103–60 days over the same periods.

This reduction in anthesis duration coincided with a marked increase in mean temperature during the pre-anthesis phase. Mean anthesis temperature increased by approximately 5.9 °C at Islamabad (reaching ~ 20.1 °C) and by 7.0 °C at Chakwal (reaching ~ 20.0 °C) between 1980 and 2020, supporting the observed acceleration in phenological development.

A similar declining trend was observed for days to maturity (DTM) at both locations. At Islamabad, DTM decreased from a maximum of 163 days in 1980–81 to 93 days in 2020–21, while at Chakwal it declined from 155 to 85 days over the same period. Regression analysis indicated a significant negative relationship between DTM and year at both Islamabad (R² = 0.72, *p* < 0.01) and Chakwal (R² = 0.72, *p* < 0.01). Decadal trends further showed a progressive shortening of maturity duration, with DTM ranges narrowing from 163 to 135 days (1980–1990) to 114–93 days (2010–2021) at Islamabad and from 155 to 127 to 131–96 days at Chakwal. Mean seasonal temperatures during the maturity phase (February–April) ranged from 10.5 to 24.8 °C at Islamabad and 11.0–25.0 °C at Chakwal.

Overall, these results demonstrate a statistically significant reduction in wheat phenological duration at both sites over the past four decades. The consistent shortening of days to anthesis and maturity is closely associated with rising seasonal temperatures, indicating increased thermal stress and accelerated crop development under ongoing climate warming.

### Relationship between growing degree days and crop phenology (DTA and DTM)

**Fig. 4:**
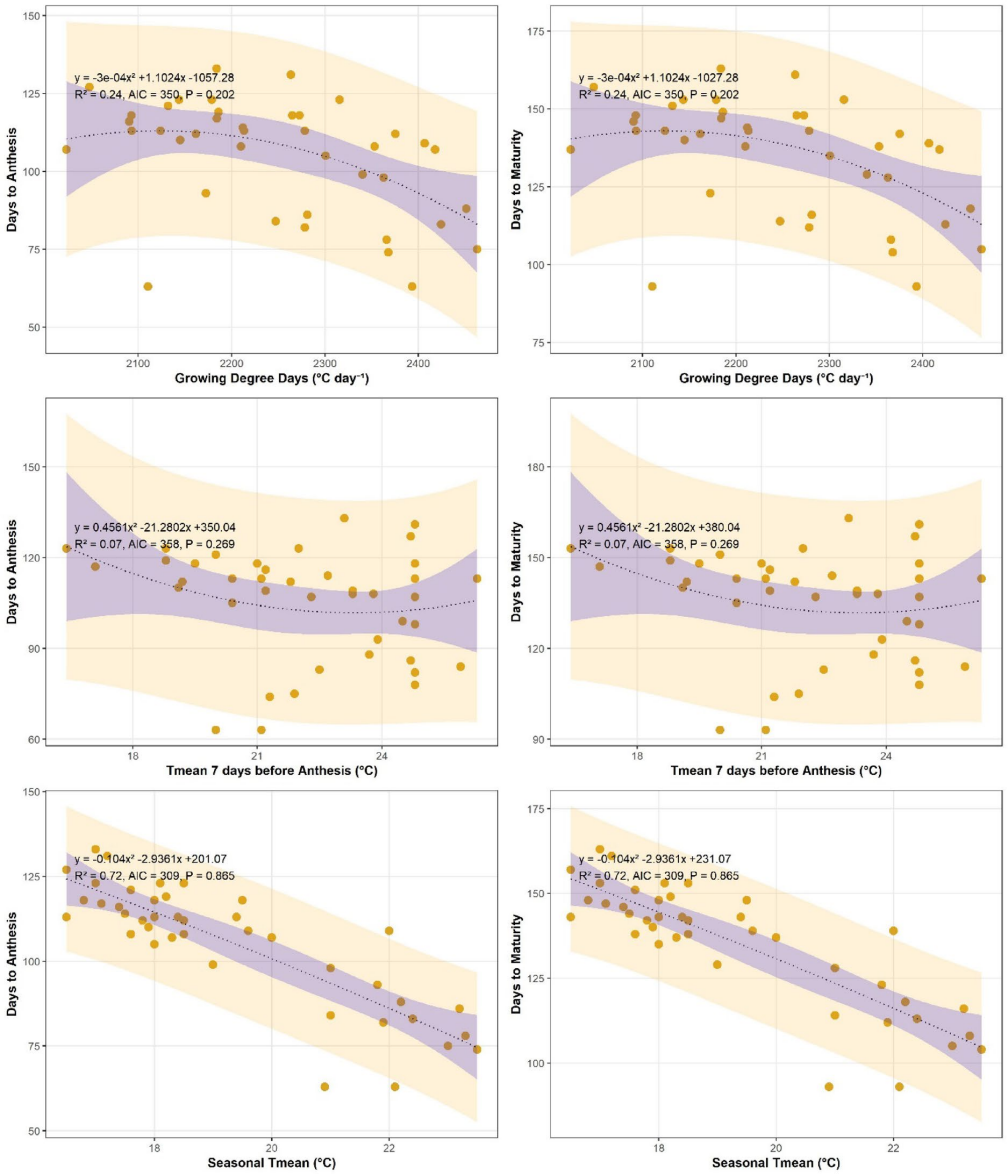
Relationship of wheat crop phenology (Days to anthesis and days to maturity) with growing degree days (^o^C Day^− 1^), T_mean_ 7 days before Anthesis (^o^C) and Seasonal temperature at Islamabad.

**Fig. 5:**
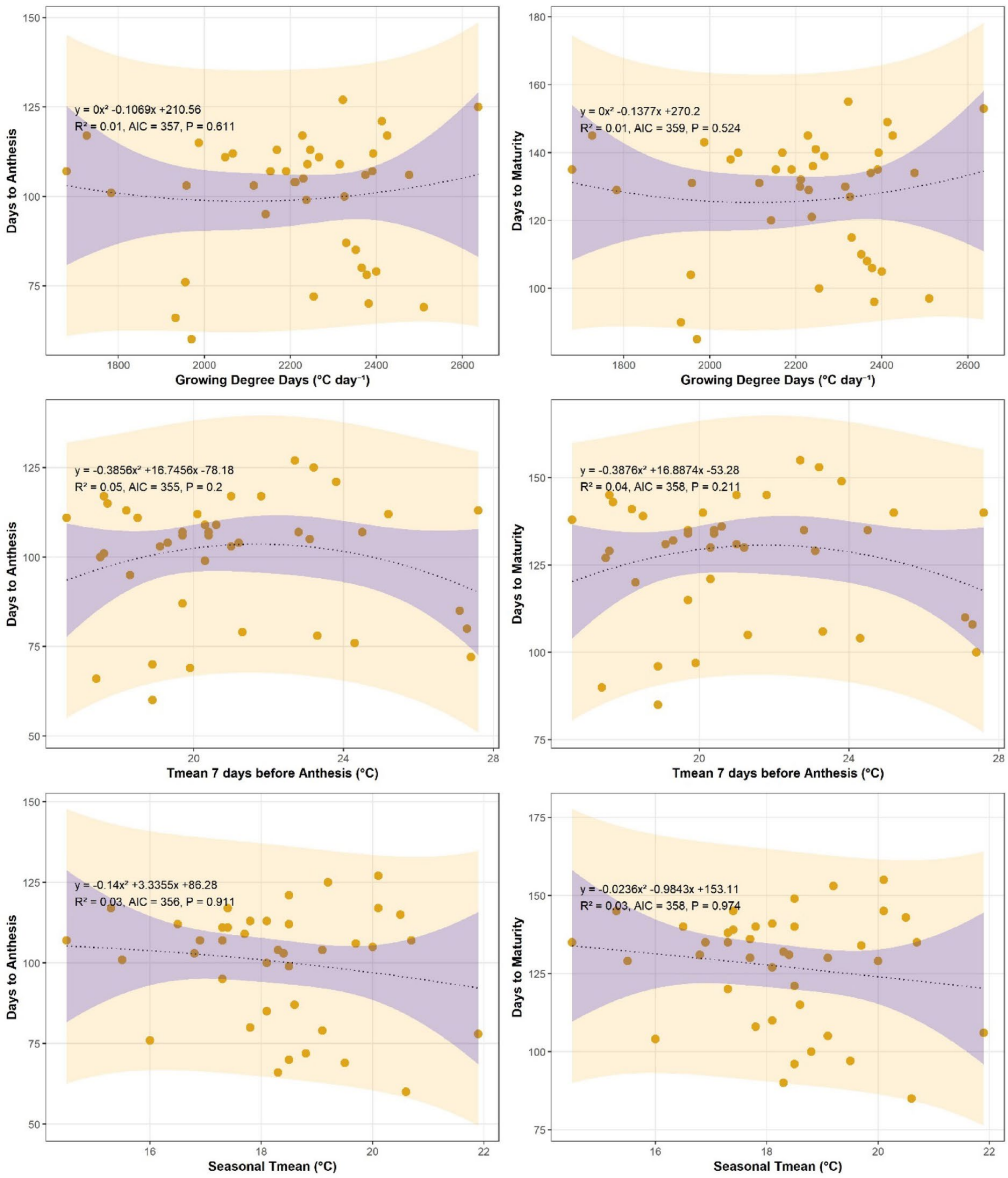
Relationship of wheat crop phenology (Days to anthesis and days to maturity) with growing degree days (^o^C Day^− 1^), T_mean_ 7 days before Anthesis (^o^C) and Seasonal temperature at Chakwal.

The relationship between growing degree days (GDD) and wheat phenology showed clear and significant trends at both study locations (Figs. 4 and 5). Increasing GDD accumulation was associated with a reduction in days to anthesis (DTA), indicating accelerated phenological development under higher thermal regimes. At Islamabad, the highest DTA values occurred under lower GDD accumulation, while minimum DTA coincided with maximum heat unit accumulation. A similar but weaker trend was observed at Chakwal, where the relationship between DTA and GDD was more linear. Regression analysis showed that at Chakwal, DTA exhibited a weak polynomial relationship with GDD (y = 3E − 05x² − 0.1069x + 210.56, R² = 0.11, *p* < 0.01), whereas at Islamabad a stronger negative relationship was observed (y = − 0.0003x² + 1.1023x − 1057.1, R² = 0.24, *p* < 0.01), indicating greater sensitivity of phenology to heat accumulation at Islamabad.

A similar response pattern was observed for days to maturity (DTM). Increasing GDD accumulation, particularly under warming and late-sown conditions, resulted in a significant shortening of maturity duration at both sites. Crops exposed to higher heat units over a shorter period reached maturity earlier, thereby reducing DTM. Regression analysis confirmed a significant negative relationship between GDD and DTM at Chakwal (y = 3E − 05x² − 0.1377x + 270.2, R² = 0.12, *p* < 0.01) and Islamabad (y = − 0.0003x² + 1.1023x − 1027.1, R² = 0.25, *p* < 0.01). The stronger response at Islamabad again highlights site-specific differences in thermal sensitivity.

In summary, increasing GDD accumulation significantly accelerated wheat phenological development, reducing both days to anthesis and maturity at both locations. These results demonstrate that rising temperatures and excessive heat unit accumulation under climate warming are key drivers of shortened crop duration, particularly in more temperature-sensitive environments.

### Average seasonal temperature pre anthesis and maturity

The effect of average temperature during the critical pre-anthesis period (seven days before anthesis) on wheat phenology is presented in Figs. 4 and 5 for both study locations. Results indicate that increasing pre-anthesis temperature significantly accelerated crop development, leading to earlier occurrence of days to anthesis (DTA). At Islamabad, a significant negative relationship was observed between pre-anthesis temperature and DTA (y = 0.4537x² − 21.149x + 348.29, R² = 0.16, *p* < 0.01), while a similar trend was found at Chakwal (y = − 0.3839x² + 16.657x − 77.093, R² = 0.24, *p* < 0.01). These results confirm that temperature increases immediately preceding anthesis shorten the duration to flowering, potentially constraining the subsequent grain-filling period.

Pre-anthesis temperature also exerted a significant influence on days to maturity (DTM). At Islamabad, DTM decreased with increasing temperature seven days before anthesis (y = 0.4537x² − 21.149x + 378.29, R² = 0.12, *p* < 0.01), and a comparable response was observed at Chakwal (R² = 0.12, *p* < 0.01). This indicates that thermal stress during the late vegetative to early reproductive stage has lasting effects on overall crop duration. The response of wheat phenology to mean seasonal temperature further highlighted the sensitivity of both DTA and DTM to warming conditions. At Islamabad, rising seasonal temperatures resulted in a significant reduction in DTA (y = 0.0751x² − 13.591x + 323.53, R² = 0.25, *p* < 0.01) and DTM (y = 0.0751x² − 13.591x + 353.53, R² = 0.26, *p* < 0.01). A similar response pattern was observed at Chakwal, where increasing seasonal temperature significantly reduced DTA (y = − 0.1523x² + 3.7682x + 82.511, R² = 0.27, *p* < 0.01) and DTM (y = − 0.0344x² − 0.6072x + 149.86, R² = 0.26, *p* < 0.01).

In summary, increases in both pre-anthesis and mean seasonal temperatures significantly shortened wheat phenological duration at both sites. These findings demonstrate that warming during critical growth stages accelerates crop development, reducing the time available for grain filling and potentially contributing to yield decline under climate change conditions.

### Grain yield in relation to thermal indices and phenological traits at Islamabad and Chakwal

**Fig. 6:**
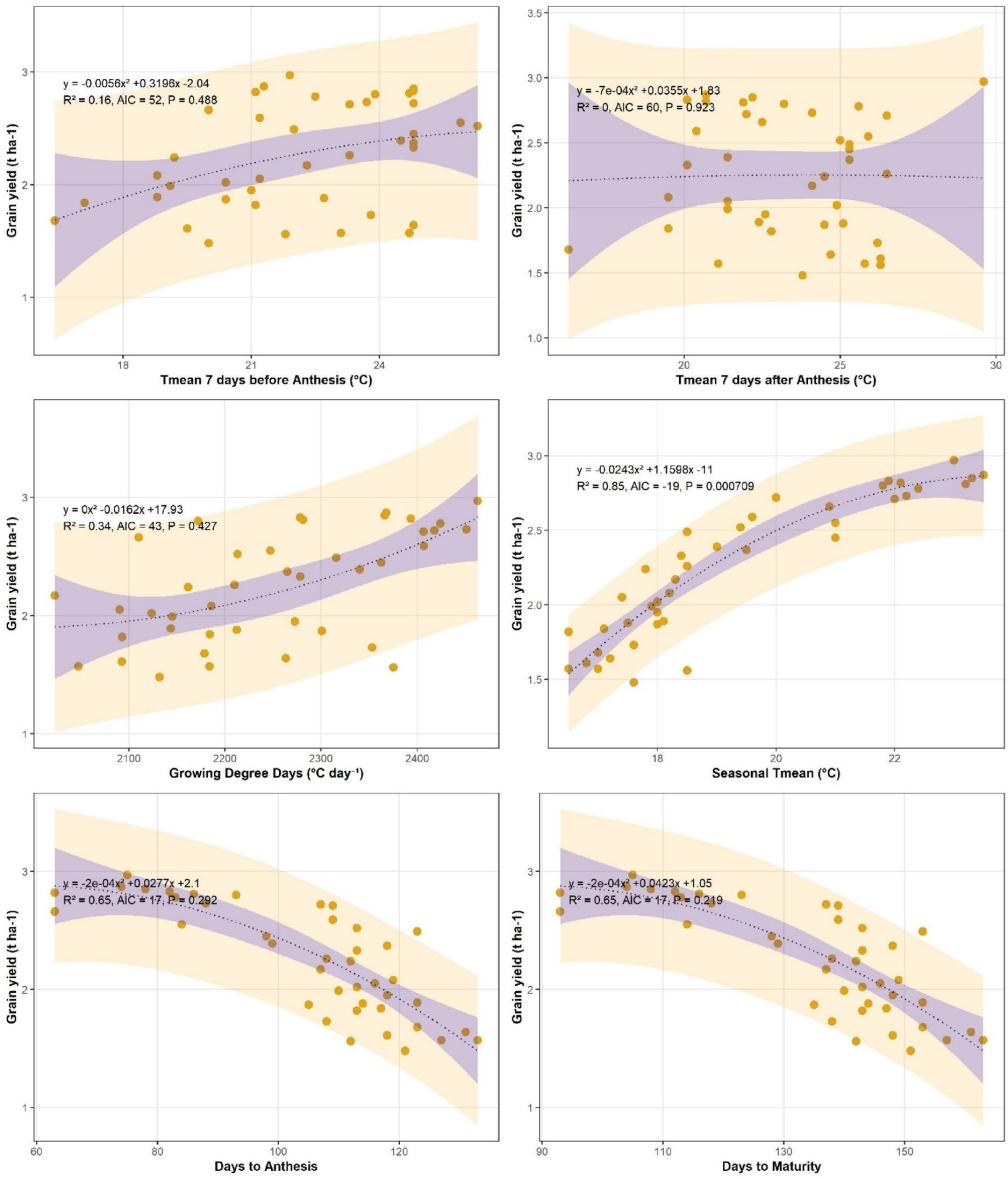
Trend of wheat grain yield (t ha^− 1^) with growing degree days (^o^C Day^− 1^), T_mean_ 7 days before Anthesis (^o^C), T_mean_ 7 days after Anthesis (^o^C), seasonal mean temperature, days to anthesis and days to maturity at Islamabad.

**Fig. 7:**
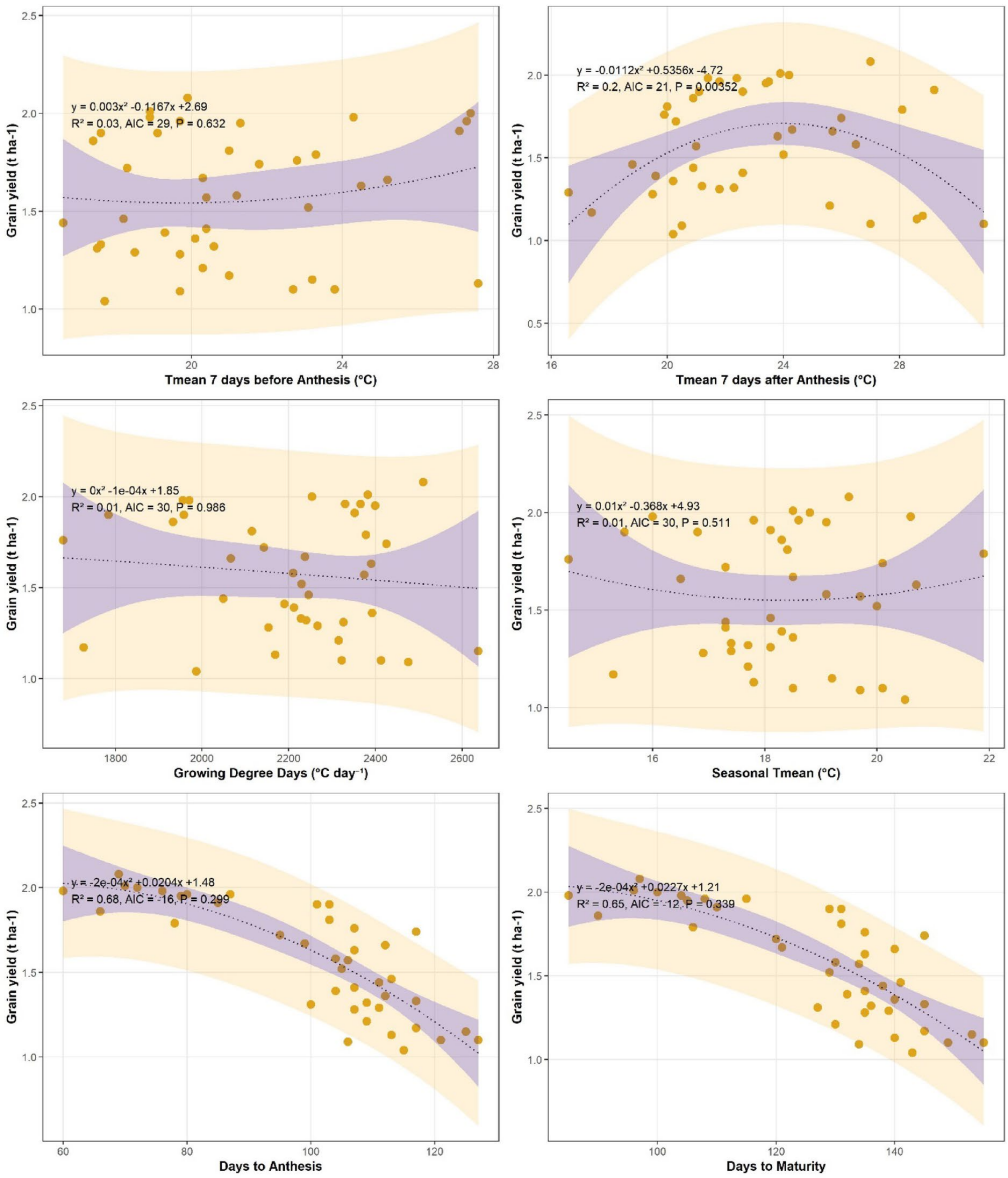
Trend of wheat grain yield (t ha^− 1^) with growing degree days (^o^C Day^− 1^), T_mean_ 7 days before Anthesis (^o^C), T_mean_ 7 days after Anthesis (^o^C), seasonal mean temperature, days to anthesis and days to maturity at Chakwal.

Grain yield showed a consistent negative association with growing degree days (GDD) at both locations, indicating that increased heat accumulation adversely affected wheat productivity (Figs. 6 and 7). At Islamabad, grain yield declined with increasing GDD, as described by the quadratic regression equation *y = 4E − 06x² − 0.0161x + 17.899* (R² = 0.34, *P* < 0.01). A similar negative relationship was observed at Chakwal, although with a slightly lower explanatory power (*y = − 1E − 08x² − 0.0001x + 1.9221*, R² = 0.28, *P* < 0.01). Overall, higher thermal accumulation during the season reduced grain yield at both sites.

Mean temperature during the critical pre-anthesis period (7 days before anthesis) exhibited a quadratic relationship with grain yield. At Islamabad, yield increased up to an optimum temperature and declined thereafter (*y = − 0.0055x² + 0.3145x − 1.9777*, R² = 0.17, *P* < 0.01). A comparable negative response beyond the optimum temperature was also observed at Chakwal (Fig. 7). Similarly, mean temperature during the post-anthesis period (7 days after anthesis) showed a negative association with grain yield, indicating sensitivity of grain filling to elevated temperatures. These results suggest that short-term temperature extremes around anthesis negatively influence wheat yield.

In contrast, long-term seasonal mean temperature exhibited a generally positive relationship with grain yield at both locations, although the strength of the association was moderate. At Islamabad, the relationship was described by *y = − 0.0246x² + 1.1416x − 10.272* (R² = 0.22, *P* < 0.01), while at Chakwal a similar trend was observed with lower explained variance (*y = 0.0105x² − 0.3872x + 5.1116*, R² = 0.18, *P* < 0.01). This indicates that moderate increases in seasonal temperature may favour yield, provided critical thresholds are not exceeded.

Phenological traits showed strong negative relationships with grain yield at both sites. At Islamabad, days to anthesis were negatively associated with grain yield (*y = − 0.0002x² + 0.0275x + 2.1051*, R² = 0.65, *P* < 0.01), while a similar trend was observed for days to maturity (*y = − 0.0002x² + 0.042x + 1.0621*, R² = 0.65, *P* < 0.01; Fig. 6). At Chakwal, days to anthesis (*y = − 0.0002x² + 0.0207x + 1.4686*, R² = 0.68, *P* < 0.01) and days to maturity (*y = − 0.0002x² + 0.0231x + 1.1897*, R² = 0.65, *P* < 0.01) also showed strong negative associations with grain yield (Fig. 7). These findings highlight that prolonged crop duration under warmer conditions leads to yield penalties at both locations.

[Insert Figs. 6 and 7 here]

### DSSAT model performance in simulating wheat phenology and grain yield at Islamabad and Chakwal

**Fig. 8:**
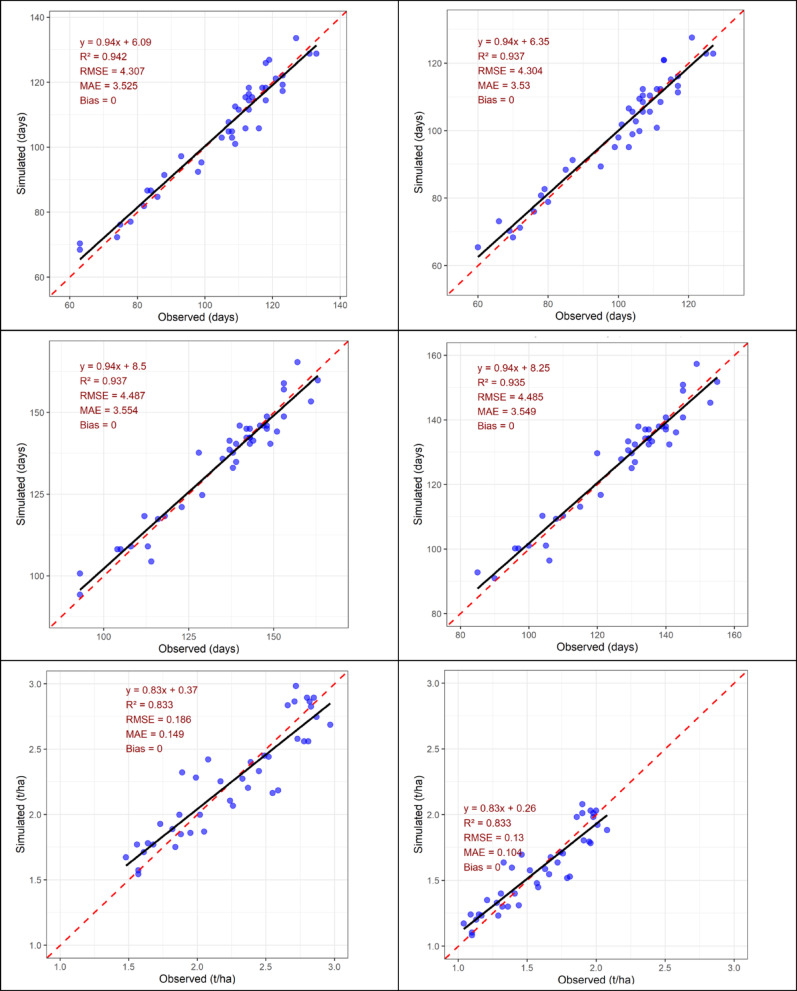
Observed vs. simulated days to anthesis (top panels), days to maturity (middle panels) and wheat grain yield (t ha^− 1^) at Islamabad (Left three panels) and Chakwal (Right three panels).

The performance of the DSSAT model was evaluated by comparing observed and simulated days to anthesis (DTA), days to maturity (DTM), and grain yield at Islamabad and Chakwal (Fig. 8). At Islamabad, the model showed very high accuracy in simulating wheat phenology. Observed and simulated DTA values were in close agreement, with a coefficient of determination (R²) of 0.94, RMSE of 3.02 days, MAE of 2.53 days, and negligible bias, indicating minimal deviation in predicting anthesis. Similarly, DTM was simulated with high precision (R² = 0.97, RMSE = 4.46 days, MAE = 3.35 days, Bias = 0.36), demonstrating the model’s robustness in representing crop developmental duration. These results confirm the strong reliability of DSSAT in simulating wheat phenology at Islamabad.

Grain yield simulation at Islamabad also showed good agreement between observed and simulated values, with R² = 0.83, RMSE = 0.188 t ha⁻¹, MAE = 0.106 t ha⁻¹, and zero bias, indicating accurate yield prediction with low error. Overall, the model effectively captured yield variability under Islamabad conditions.

At Chakwal, the model performance was slightly lower but remained satisfactory. Simulated DTA exhibited a strong correlation with observations (R² = 0.87), although error values were marginally higher (RMSE = 3.20 days, MAE = 3.53 days, Bias = 0). Predictions of DTM were consistent across sites, with R² = 0.95, RMSE = 4.46 days, MAE = 3.46 days, and no bias, confirming stable model performance for maturity estimation. Thus, DSSAT reliably simulated phenological stages at Chakwal despite increased variability.

Grain yield simulation at Chakwal showed reasonable agreement with observed data (R² = 0.83, RMSE = 0.198 t ha⁻¹, MAE = 0.116 t ha⁻¹, Bias = 0), although greater scatter in data points suggested comparatively higher uncertainty than at Islamabad. Nevertheless, yield predictions remained within acceptable accuracy limits. Overall, the DSSAT model demonstrated strong predictive capability for wheat phenology and grain yield at both locations, as reflected by high R² values (0.83–0.97) and low error statistics. Model performance was consistently better at Islamabad than at Chakwal, likely due to differences in environmental conditions and soil fertility between the two sites. In conclusion, DSSAT proved to be a reliable tool for simulating wheat growth and yield under contrasting agro-climatic conditions.

### Sensitivity analysis of wheat grain yield to CO₂ concentration and temperature

**Fig. 9:**
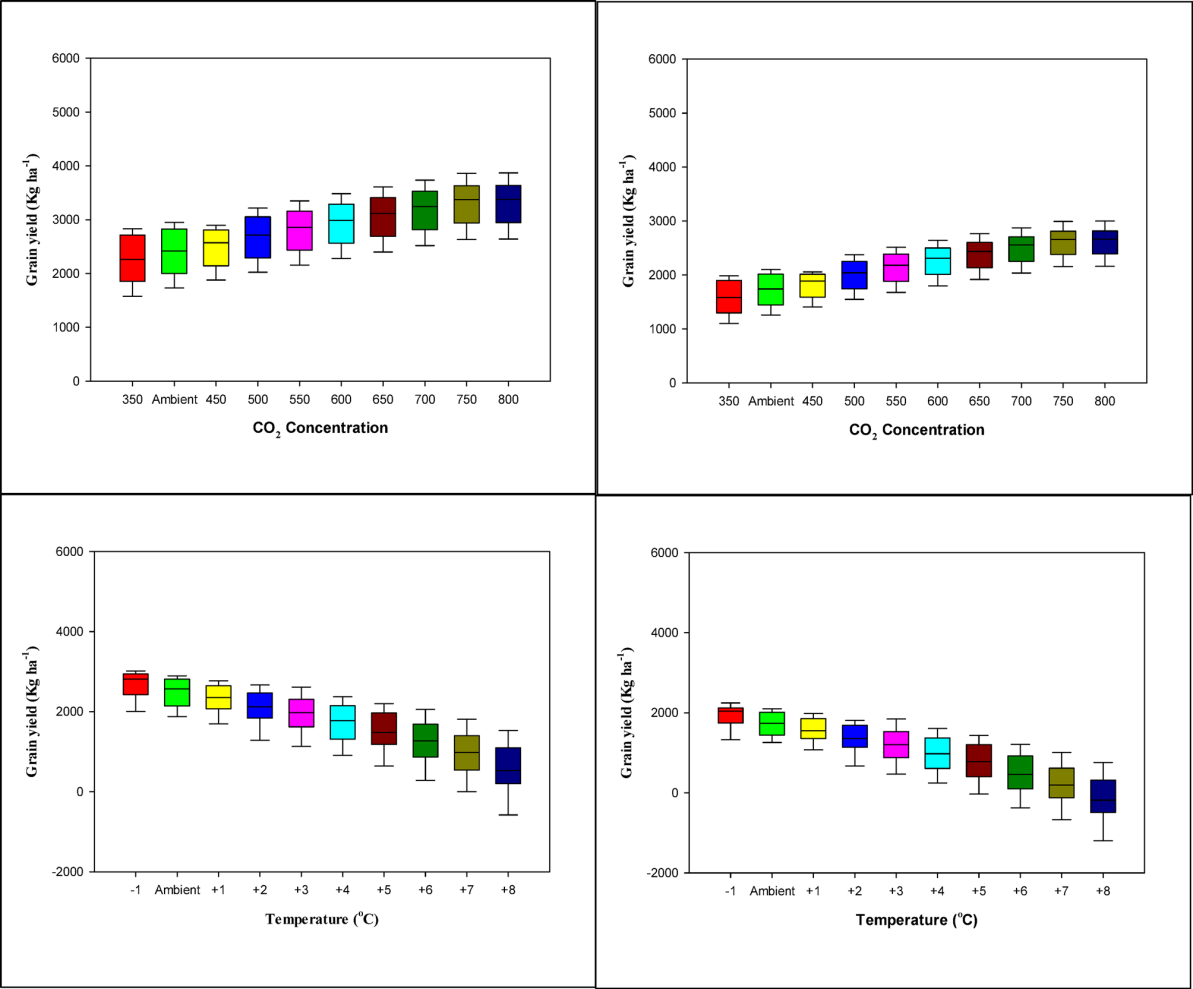
Sensitivity analysis of the wheat grain yield (kg ha-1) at various (a, b) CO_2_ concentration (ppm) and (c, d) change in temperature (^o^C) at Islamabad (a, c) and Chakwal (b, d). The boxplots reflect the variation of the simulated yields in each case of CO_2_ or temperature condition.

Sensitivity analysis showed contrasting responses of wheat grain yield to changes in atmospheric CO₂ concentration and temperature at both Islamabad and Chakwal (Fig. 9). Increasing CO₂ levels from 350 to 800 ppm resulted in a progressive increase in grain yield, ranging from 6 to 9% at Islamabad and 5–8% at Chakwal. This yield enhancement reflects the positive physiological effects of elevated CO₂ on photosynthesis and water-use efficiency. Overall, elevated CO₂ exerted a beneficial but moderate influence on wheat yield at both sites.

In contrast, grain yield exhibited a strong negative response to increasing temperature. A rise of 1 °C above ambient temperature reduced yield by approximately 4.5% (≈ 0.18 t ha⁻¹) at Islamabad and 6% (≈ 0.24 t ha⁻¹) at Chakwal. Yield losses intensified under extreme warming scenarios, exceeding 35–40% at temperature increases of + 7 to + 8 °C, indicating high sensitivity of wheat to heat stress, particularly during reproductive stages. These results demonstrate that temperature increases pose a substantial risk to wheat productivity.

Although elevated CO₂ partially mitigated yield reductions under warming conditions, the compensatory effect was insufficient to offset the negative impact of higher temperatures. This response is consistent with accelerated growing degree day accumulation and shortened grain-filling duration under heat stress. In summary, future warming is likely to outweigh the benefits of CO₂ fertilization, highlighting the need for adaptive strategies such as optimized sowing time and heat-tolerant cultivars to sustain wheat productivity in the rainfed Pothwar region.

### Temperature impact on wheat flowering under varying sowing date scenarios

Long-term analysis showed that wheat phenology and yield were strongly influenced by sowing date–induced temperature variations at both Islamabad and Chakwal (Table 4). Mean temperature (Tmean) during flowering increased progressively with delayed sowing from 15 October to 24 November. At Islamabad, Tmean at flowering increased from 11.06 °C for the earliest sowing to 16.61 °C for the latest sowing, while at Chakwal it increased from 10.24 °C to 15.92 °C over the same period. This corresponded to a significant rise in Tmean of 33.41% at Islamabad and 35.67% at Chakwal between the earliest and latest sowing dates. These results indicate that delayed sowing substantially exposes wheat to higher temperatures during flowering.

**Table 4:** Sowing date scenarios and wheat flowering duration at Islamabad and Chakwal.

Islamabad					
Date of Sowing	Days to Anthesis	Date of Anthesis	Flowering duration	Long term Average Temperature (^o^C)	Yield loss %
15-Oct	105	27-Jan	10 Days	11.06	Zero
25-Oct	105	07-Feb	10 Days	11.89	Zero
04-Nov	105	17-Feb	10 Days	13.61	9%
14-Nov	105	27-Feb	10 Days	14.57	15%
24-Nov	105	09-Mar	10 Days	16.61	27%

Chakwal					
Date of Sowing	Days to Anthesis	Date of Anthesis	Flowering duration	Long term Average Temperature (^o^C)	Yield loss %
15-Oct	100	22-Jan	10 Days	10.24	Zero
25-Oct	100	01-Feb	10 Days	11.19	Zero
04-Nov	100	11-Feb	10 Days	12.45	0.25%
14-Nov	100	21-Feb	10 Days	13.53	10%
24-Nov	100	03-Mar	10 Days	15.92	25%

The first two sowing dates experienced relatively favourable thermal conditions during anthesis, whereas later sowings were subjected to elevated temperatures that resulted in notable yield reductions. At Islamabad, yield losses for the third, fourth, and fifth sowing dates were 9%, 15%, and 27%, respectively. At Chakwal, corresponding yield losses were 0.25%, 10%, and 25%. This trend demonstrates that late sowing increases heat stress during flowering, leading to progressive yield penalties at both sites.

Analysis of long-term rainfall patterns further revealed variability in precipitation distribution across crop growth stages (Table 5). During 1980–1990, Islamabad received the highest rainfall at the vegetative stage (828 mm) and the lowest at the reproductive stage (268 mm), whereas Chakwal received maximum rainfall during the reproductive stage (180 mm) and minimum during the vegetative stage (61 mm). Similar patterns persisted in subsequent decades, although the magnitude of rainfall fluctuated across growth stages. In the most recent decade (2011–2020), the highest rainfall at the reproductive stage was recorded at both sites (371 mm at Islamabad and 241 mm at Chakwal), suggesting a shift in seasonal precipitation dynamics. Overall, changing rainfall distribution, combined with rising temperatures under late sowing, may further influence wheat flowering and yield stability.

**Table 5:** Decadal (1980–2020) average rainfall at Islamabad and Chakwal during vegetative and reproductive stage.

Islamabad			Chakwal		
Decade	Vegetative	Reproductive	Decade	Vegetative	Reproductive
1980–1990	828	268	1980–1990	61	180
1991–2000	717	170	1991–2000	58	149
2001–2010	67	143	2001–2010	50	138
2011–2020	111	371	2011–2020	85	241

### Correlation analysis

**Fig. 10:**
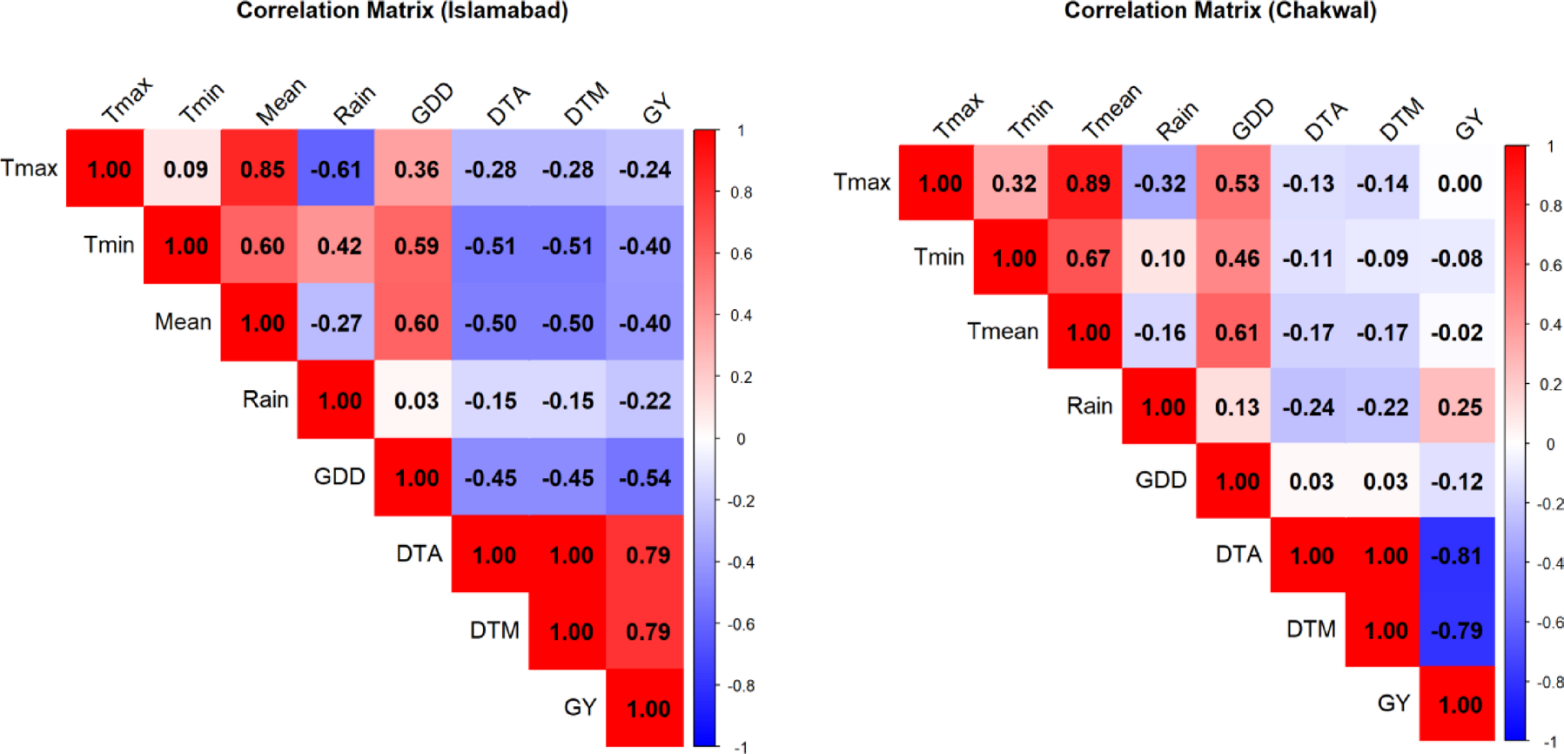
Correlation matrix of agro-metrological parameters with phenology and yield at Islamabad and Chakwal (Where T_max_: Maximum temperature, T_min_: Minimum Temperature, T_mean_: Average temperature, GDD: Growing degree days, DTA: Days to anthesis, DTM: Days to maturity, GY: Grain yield).

Correlation analysis revealed predominantly negative relationships among temperature variables, phenological traits, and grain yield at both study sites (Fig. 10). Increases in both maximum (Tmax) and minimum temperature (Tmin) were negatively correlated with grain yield, days to anthesis (DTA), and days to maturity (DTM), indicating adverse effects of elevated temperatures on wheat development and productivity at Islamabad and Chakwal. Overall, higher temperatures consistently reduced phenological duration and yield across sites.

Rainfall also showed negative associations with phenological traits at Chakwal, where increased precipitation was correlated with reduced DTA and DTM. At Islamabad, rainfall exhibited negative correlations not only with DTA and DTM but also with grain yield, suggesting that higher or poorly distributed rainfall may negatively influence both crop development and yield formation. These results highlight site-specific rainfall effects on wheat phenology and yield.

Growing degree days (GDD) were negatively correlated with grain yield at Chakwal, emphasizing the yield-reducing effect of cumulative heat exposure. At Islamabad, GDD showed negative correlations with grain yield as well as with DTA and DTM, indicating a broader influence of heat accumulation on crop growth duration and yield potential. Thus, increased thermal accumulation shortened crop duration and reduced yield, particularly at Islamabad.

In summary, the correlation patterns demonstrate strong linkages between climatic variables and wheat growth, with rising temperatures, accumulated heat units, and variable rainfall exerting negative effects on phenology and grain yield. These findings confirm the sensitivity of wheat production to climatic variability at both locations.

## Discussions

The analysis conducted over the period from 1980 to 2020 underlines the substantial impact of climatic changes on wheat crop phenology and yield at both Islamabad and Chakwal. The average temperature during wheat growing seasons have been increased by approximately 1.5 °C at Islamabad and 1 °C at Chakwal over the 41 years period. This trend is consistent with global observations, as evidenced by earlier studies^44–47^ who reported a significant rise in both maximum (T_max_) and minimum (T_min_) temperature. The warming pattern reported in this study is a part of a wider, long-term climatic change that is changing the productivity of agriculture globally. An example is the Northern China Plain, major wheat growing zone, where T_min_ have been increased at a rate of 0.05 °C per year and T_max_ by 0.03 °C per year over similar multidecadal timeframes^14,48,49^. This supports the finding that the warming trends in Pakistan are not a localized event, but are entrenched in a global trend of climate change, which has been majorly fuelled by anthropogenic emission of GHGs. These thermal changes have far reaching consequences, especially on crops that are sensitive to temperature such as wheat. Any rise in temperatures above optimum levels causes an acceleration in crop development, shortening of growth periods, and an increase in stress during sensitive stages of crop development, e.g., anthesis and grain filling, all of which can reduce yield potential^50^. Monitoring and quantification of these temperature trends should thus be sustained in the future to generate agronomic strategies to adapt and inform the regional agricultural policies^51^. The consistency of these trends across different geographical locations underscores the global scale of climate change and its potential to influence agricultural systems worldwide. The concept of a “ripple effect” as mentioned by Gule, et al^52^. suggests that climatic changes in one part of the globe can have far-reaching impacts on other regions. This interconnectedness highlights the importance of considering global climatic trends when assessing the sustainability and productivity of agricultural practices^53^. Given the pivotal role of wheat in global food security, the observed climatic changes and their impacts on wheat phenology and yield point to the urgent need for adaptive strategies^54^. These strategies could include the development of climate-resilient crop varieties, the adoption of innovative agricultural practices to manage the risks associated with climate variability, and policies aimed at mitigating the effects of climate change on agriculture^55^. Overall, the evidence suggests that addressing the challenges posed by climate change to agriculture requires a coordinated global response, leveraging scientific research, policy-making, and practical interventions to ensure the resilience and sustainability of food systems worldwide^56–60^.

The 1.5 °C rise in mean temperature during the wheat growing season has significantly resulted to the reduced phenology and yield of wheat crop. DTA and DTM were decreased significantly by this thermal increase (percentage change of 44.51 and 43.00, respectively). The results are in line with the results obtained by^61^, who reported phenological reduction under the condition of increasing temperature. These decreases in DTA and DTM are signs that there is a narrowing of developmental window that may inhibit vegetative growth, decrease biomass accumulation and ultimately yield. Our results are in line with previous conclusions made by^62^, who stressed that high temperatures hasten the growth of crops, which in most cases compromises the grain filling time and reduces yield. These findings also echo the work by Verón, et al^63^. who concluded that an increase of 1 °C in the average temperature (pre- and post-anthesis) led to large yields losses. Their research included the fact that even small temperature increases can aggravate heat stress at sensitive growth stages resulting in impaired reproductive growth and a smaller grain size. Notably, the level of the changes in the phenology in the present research indicates how sensitive wheat is to thermal stress, particularly when warming is in association with critical periods of growth. These patterns are similar in other geographic settings (e.g. South Asia, South America, and East Asia), which further contributes to the applicability of the results globally and highlights the necessity of adaptive strategies to reduce climate-driven yield punishment, e.g. altered sowing dates or cultivars that are heat-tolerant. Moreover, the fact that increasing temperatures cause shortening of wheat phenology i.e. DTA and DTM has been supported by the previous research. A number of researchers have documented that high temperatures have always resulted in an earlier appearance of the crucial events in the life cycle of wheat such as anthesis, physiological maturity^22,64,65^. These results affirm the phenological sensitivity of wheat to temperatures and the acceleration of the phenology due to warming. Research by Tao, et al^66^. revealed reductions in DTA at 43 out of 108 investigated stations, along with significant advances in DTM at 41 out of 109 stations, further illustrating the impact of temperature on wheat phenology. Croitoru, et al^67^. documented a rise in temperature ranging from 0.06 °C to 0.3 °C per decade, resulting in decreased DTA by 0–3 days per decade and DTM by 1–3 days per decade. These findings collectively underscore the profound influence of temperature increases on wheat phenology and yield, emphasizing the importance of understanding and addressing the impacts of climate change on agricultural systems. It is to be noted that this study was carried out on only one type of wheat which is the most common to grow on rainfed conditions in the Pothwar region. This method was used to guarantee that the findings are directly applicable to most farmers, yet it does restrict the possibilities of examining any potential varietal distinctions. It has been demonstrated before that cultivars vary vastly in their thermal time requirements, days filled, and tolerance to terminal heat stress^68^. Lack of varietal comparisons in our data set implies that the observed shortening in phenology of growth and yield decreases may be mostly due to the sensitivity of the local cultivar predominantly used. Multi-cultivar trials and simulation experiments should however be included in future work to assess the performance of recently released and climate-resilient wheat lines under warming conditions, which would form a more robust foundation on which to advise specific adaptation strategies.

Extreme temperature is widely known to be among the most severe abiotic stress factors that inhibit plant development, growth and eventual production, especially in cereal crops^69^. Thermal stress has shown negative impact during reproductive stage particularly in temperate and subtropical agro-climates. It has been reported in many studies^70–73^ that high temperatures are able to cause earlier anthesis. These phenological shifts in the present study depicted considerable yield losses, 39% yield loss in wheat at Islamabad, and a 25% yield loss in wheat at Chakwal, associated with the observed temperature rise during study period. Such losses in yield have been observed in earlier studies. They pointed that even modest increase in mean temperature during the reproductive period can induce considerable yield losses, especially in non-irrigated or marginal conditions^74,75^. These findings underscore the susceptibility of wheat production systems in semi-arid areas to an increase in temperature, especially at critical growth processes. They also emphasize on the urgent need to implement adaptive mechanisms which include development and use of heat tolerant cultivars, change in dates of sowing and better soil and water management methods to counter the negative effects of terminal heat stress. Although the main aim of this study was to evaluate the impact of increase in temperature, it is also necessary to note the possible effect of the distribution patterns of rainfall on the yield of wheat in rainfed Pothwar region. The seasonal totals on their own fail to fully describe the crop-climate interactions, since the timing, frequency, and intensity of rainfall tend to affect soil moisture availability at the key phenological stages. An example is the limitation of tiller growth and biomass development by lower or delayed rains in the early vegetative stage, and the worsening of the adverse impact of high temperatures by water shortages during anthesis and grain filling, which increase maturity, and yield potential^76^. On the other hand, intense rainfall events over a short span can result in runoff losses and soil erosion instead of productive recharge of moisture. The net impact of warming and unpredictable distributions of rainfall could thus compound risks to wheat productivity in rainfed regions. Further studies are recommended to combine temperature and rainfall distribution versions, with process-based models, including DSSAT and APSIM, to offer more sound information on how the climate variability plays out with the wheat phenology and productivity in semi-arid environments.

DSSAT model was highly successful in the simulation of wheat phenology and grain production at both Islamabad and Chakwal even though differences based on location were also observed (Fig. 8). Phenological stages, e.g. DTA and DTM, were also simulated with high precision, which is consistent with the past research findings that phenology is typically the most reliably simulated aspect of wheat models^77^. The yield forecasts were also fairly precise (R^2^ = 0.83), yet more variable than phenology, as they are the result of the intricate interaction of environmental pressures, soil heterogeneity, and management strategies, which crop models have not always been able to explain adequately^78^. The slightly improved performance at Islamabad over Chakwal can be explained by the more stable environmental conditions, closer correspondence with the calibration data or reduced variability of the stress factors, but the increase of the scatter at Chakwal can be explained by the localized soil and climate heterogeneity, unaccounted stress of nutrients or variability of the measurement. Such problems with prediction of yields have also been observed in multi-site tests in which model performance suffered when under stressful or unusual conditions^79^. These findings highlight the fact that the model can be safely used to test the scenario of wheat production in such areas, though one should pay close attention to the uncertainties. Further development should aim at improving site-specific soil and nutrient parameters, the incorporation of improved stress-response functions, and the selection of an ensemble modeling approach, which is proven to increase the reliability in different settings^80^. Process-based crop models are an important step to improving the predictive ability of climate impact assessments. The DSSAT model performed well by simulating wheat phenology and yields at Islamabad and Chakwal with a high level of agreement between the observed and simulated values (R^2^ = 0.83). These findings coincide with previous publications according to which phenological stages can be considered the most consistently simulated element in wheat models^17,81^. By combining the results of DSSAT with empirical analysis, our findings are better supported and the association between the phenological changes due to temperatures and the economic changes that could be shown to farmers is enhanced. However, as reported earlier^82^, no single crop model can adequately reflect the complex interactions involved in soil, climate, and management. Therefore, an ensemble modeling strategy (e.g., DSSAT, APSIM, CropSyst, WOFOST) could be advantageous in future studies of the area, as it has been demonstrated that it minimizes uncertainty and increases the accuracy of yield forecasting in various settings. With a mixture of trends and process-based simulations, our work provides a more robust platform to predict the effects of climate change and develop region-specific adaptation pathways.

Additionally, the increased in trend of days with high temperatures above threshold 35 °C during the maturity stage (March-April) is an important factor affecting grain sterility and seed weight. Heat stress is often a limiting parameter that cause significant reduction crop phenological period and yield^83–85^. Different observations other than our findings showed that due to rise in seasonal temperature maximum number of heat units accumulated which hasten the maturity due to which grain yield has been dropped to 1.10 t ha^− 1^ from 4.08 t ha^− 1^ at Islamabad while at Chakwal it was decreased to 0.86 t ha^− 1^ from 3.68 t ha^− 1 11^. Lobell and Field^14^ documented that mean temperature during the wheat growing season and grain yield showed negative relationship among each other. Study also proved that due to increase in mean temperature crop maturity duration decreased by 16 days from 130 which directly affect the grain yield. Similarly it has been documented that due to significant rise in temperature from 14 °C to 23 °C wheat yield has been linearly decreased 0.25 g plant ^− 1^ °C^− 1^ about 5% °C^87^ described. Furthermore, it has been noted that average increase in wheat seasonal temperature by 2 °C can reduce the grain yield by 50% ^71,88^. Thus, this reduction will cause significant economic damage to the farmers so adoption of adaptation strategies like changing sowing dates as suggested in our work should be considered to minimize the economic damage to the farming community. However, earlier studies have suggested different adaptation options e.g. heat index insurance^89^,change in sowing date and modification in the fertiliser applications^31–33,90^, optimal use of agronomic inputs^91^, availability of tolerant cultivars at field scale^92^, designing of wheat ideotypes having medium-late maturity, larger grain size and higher RUE^93^ and development of crop and region-specific adaptation strategies through use of process based models to ensure future food supply^94^. Figure 9 shows the impact of elevated CO_2_ and temperature on grain yield of wheat. Thus, it is utmost important to use adaptation strategies otherwise we will be having great economic loss. Under current government fixed rate (e.g. 3100 Pakistani Rs) of wheat price per 40 kg farmers will be having 61.62 maund (61.62 × 40 = 2464.8 kg or 2.46 t) that will generate income of 685.9 US$. However, with every1^o^C rise in temperature there is significant decline in the economic returns that shows how important it is to implement adaptation options by forcing policy makers to act now otherwise it will be too late. This sensitivity analysis (Fig. 9) shows that high CO_2_ atmosphere increases the yield of wheat because it leads to higher photosynthetic efficiency and water use, but the effect of this is overridden by the negative effect of the increasing temperature. The decrease in yield was about 4–6% per 1 °C rise in temperature which is in line with other past studies in semi-arid areas. The decrease is attributed to the increased GDD accumulation and a decrease in grain-filling period in heat stress, especially in the period of anthesis. Furthermore, an increase in temperature increases the process of evapotranspiration, restricting the soil moisture in rainfed systems such as Pothwar. These results demonstrate that CO_2_ enrichment offers partial mitigation yet temperature is the prevailing limitation so as to achieve sustainability. The relevant adaptation has to be oriented towards heat tolerant cultivars, modified sowing dates and the maintenance of soil moisture to retain productivity under the assumed warming scenarios. On the whole, this paper highlights that climate-resilient management practices are needed to counter this loss of yield and maintain wheat production in the risky rainfed areas.

Temperature played a key role in modification of plants enzymatic functions and it can cause change in crop phenology and reduced crop yield^47^. It is well known phenomenon that the duration of maturity of wheat crop is shortened by heat stress^95^. It is also proven that each degree rise in seasonal temperature during grain filling duration about three days reduction in the duration of grain filling have been observed^96^. Growing degree days concept is good indicator to determine accumulated heat unit effect on crop phenology and yield^97^. At Islamabad GDD changed from 2000 °C days^− 1^ to 2500 °C days^− 1^ during last four decades while at Chakwal it was 1679 °C days^− 1^ to 2637 °C days^− 1^. It has been observed that wheat yield highly sensitive towards temperature^9,36,98^. Further observations of conducted study documented that crop face temperature above 35 °C which induce early anthesis and maturity which directly impacted on wheat yield. Similar to our findings^99–101^reported that wheat yield has been reduced due to increase in the number of days with a temperature above 35 °C during the maturity stage^66,102–104^. reported parallel to our findings that wheat required approximately 2200 heat unit by using 4 °C as a base temperature above mention range crop development stages accelerated which turn leads to reduce yield as well as wheat growing season.

### Economic feasibility and management barriers of adaptation strategies

Findings by this study indicate that a 1.5 °C increase in seasonal temperature not only reduced wheat phenology but also resulted in a considerable yield loss of 39% and 25% at Islamabad and Chakwal respectively. Monetizing these falls in the yield would translate into monetary terms, the production of wheat at Islamabad was reduced to 1.10 t ha⁻¹ against 4.08 t ha^− 1^, at Chakwal it was reduced to 0.86 t ha^− 1^ against 3.68 t ha^− 1^. This shows 65% per hectare loss in farm income considering current government support price (3,100 PKR/40 kg, 77.5 US$ t^− 1^). These drastic cuts remind us of the economic imperative to implement adaptation measures (Table 6).

**Table 6:** Economic feasibility and adoption hindrance of the chosen wheat adaptation measures.

Adaptation option	Expected yield benefit	Estimated income benefit (per ha)*	Economic feasibility	Major adoption barriers
Sowing date adjustment	0.5–1.0 t ha⁻¹ (by avoiding terminal heat stress)	40,000–60,000 PKR (≈ 150–220 US$)	Low-cost, immediately actionable; requires only calendar shifts and management coordination	Conflicts with crop rotation calendars (e.g., rice–wheat system), limited irrigation water availability, lack of timely machinery/labor, smallholder risk aversion
Heat-resilient cultivars	1.0–1.5 t ha⁻¹ (stabilized yields under high temperatures)	80,000–120,000 PKR (≈ 280–430 US$)	High long-term return, enhances resilience against future warming	High seed costs, limited certified seed availability, weak distribution systems, farmer reluctance to replace traditional varieties, uncertain performance under heterogeneous field conditions

*Income benefits estimated at 3,100 PKR per 40 kg (≈ 77.5 US$ per ton).

Sowing date adjustments are among the most economically viable short-term solutions. According to simulation studies (such as DSSAT scenarios used in this study), a shift in sowing dates of 10–15 days could help avoid exposure to terminal heat, avoiding a yield loss of 0.5–1.0.5.0 t ha^− 1^. This equals to a possible profit of 40,000–60,000 PKR (about 150–220 US$) per hectare at the present price of wheat, with little extra expense other than better management coordination. The results are similar to those of previous reports that show that timely sowing greatly mitigates the risk of heat stress in wheat^20,68,105^. Adoption is, however, hindered by conflicting crop calendars (e.g., late rice harvest postponing wheat planting in Punjab), access to water to plant at the right time, and smallholder reliance on workforce or equipment that might not be available at the right time^106^(Table 6).

Heat resistant cultivars, however, constitute a medium to long term approach. If such cultivars could be able to stabilize yield even at 70–80% of potential in high-temperature environments, farmers would gain a 1–1.5.5 t ha^− 1^ more than current varieties. This would cost economically 80,000–120,000 PKR (= 280–430 US$) per hectare. There are other reports of similar yield stabilization in heat-tolerant varieties in South Asia^104,107^. However initial investment in certified seed (2500–4000 PKR per acre) and the requirement to change varieties every few years pose a challenge to farmers with limited resources. Additionally, lack of access to superior seed, poor extension services, and distrust toward new varieties lower the level of adoption^108^. Even when yields are reduced in a stressed climate, farmers tend to use cultivars that have been shown to work in their particular soil, water, and management environment (Table 6).

Comparatively, sowing date adjustment is low cost, actionable, immediately, and with small yield increase, as compared to resilient cultivars which is an expensive, long-term investment with big yield and income improvements. Neither of the two strategies can be adopted without facing barriers to adoption: farming systems and access to resources limit sowing date shifts, while the adoption of cultivars is hindered by limitations in the seed system, cost, and risk aversion by farmers.

Policy support and incentives are important to maximize feasibility. Barriers can be reduced with subsidies on certified seed, subsidies on mechanized sowing services, and investments in weather prediction equipment. In addition, farmer confidence in heat-resilient cultivars can be established through participatory varietal trials and demonstration plots^109^. The danger without such enabling strategies is that smallholder farmers, the most susceptible to yield losses due to climate changes, will be equally the least capable of implementing the measures necessary to protect their livelihoods.

#### Study limitations and potential future directions

Studying how climate change affects rainfed spring wheat over decades is vital for sustainable agriculture, but it comes with challenges. Data quality can vary, especially in regions with few monitoring systems, leading to uncertainties. Also, coarse data resolution might miss fine-scale climate and crop variations. Other factors like soil quality, pests, and farming practices aren’t always considered, affecting results’ accuracy. Findings from one region or time may not apply elsewhere due to different conditions and responses. Long-term studies are rare, making it hard to separate short-term fluctuations from long-term climate impacts. Predicting future effects relies on uncertain statistical models affected by emissions scenarios and internal variability.

In the years to come, there are several ways we can deepen our understanding of how climate change impacts rainfed spring wheat. Firstly, we can enhance our data collection methods by improving monitoring networks and sharing data more effectively. Additionally, employing sophisticated crop models will allow us to better grasp the intricate relationships between climate variables, soil conditions, and crop growth, including their complex interactions. By utilizing high-resolution climate and crop modeling approaches, we can capture fine-scale variations and develop tailored adaptation strategies for local conditions. Moreover, integrating factors such as soil health, agronomic practices, and socio-economic dynamics into our modeling frameworks will provide more comprehensive assessments of climate change impacts. Continuation of long-term monitoring efforts will enable us to track changes in rainfed spring wheat phenology and yield over multiple decades, facilitating more accurate trend analyses and predictive modeling^29,110–114^. Furthermore, conducting region-specific investigations will help us understand localized variations and devise region-specific adaptation strategies. Finally, we need to evaluate the risks associated with climate change impacts on rainfed spring wheat production and develop adaptive strategies, such as crop diversification, improved irrigation management, and breeding for climate resilience. By addressing these challenges and pursuing these future directions, we can advance our understanding of the long-term impacts of climate change on rainfed spring wheat production and inform policies and practices for sustainable agriculture in a changing climate.

## Conclusion

In conclusion, our comprehensive study utilizing long-term datasets of phenology, yield, and meteorological observations has shed light on the significant impact of rising temperatures on wheat phenology and yield at both Islamabad and Chakwal sites over the past four decades (1980–2020). The observed increase in temperature during the wheat growing season has led to reductions in days to anthesis (DTA), days to maturity (DTM), and ultimately, yield. A continued temperature rise may disrupt wheat vernalization, negatively affecting phenology and yield. The increasing trend of days with high temperatures above the threshold of 35 °C during the maturity stage (March-April) is also identified as a crucial factor affecting grain yield. To mitigate the adverse effects of rising temperatures on wheat phenology and yield, we propose the adoption of sowing date manipulation as an adaptation strategy. Specifically, we recommend shifting the sowing date to between 1 st and 15th October, coupled with the utilization of versatile (i.e. early, medium and late maturing) and heat-resistant germplasm.

## Supplementary Information

Below is the link to the electronic supplementary material.

Supplementary Material 1

## Data Availability

The datasets used and/or analysed during the current study available from the corresponding author on reasonable request.

## Abbreviations

GDD: Growing degree days
SD: Sowing date
DTA: Days to anthesis
DTM: Days to maturity
IPCC: Intergovernmental Panel on Climate Change
