# Supplementary figures and images for: Warming trends and shortened growing seasons: integrating four decades of observations and model simulations to develop wheat adaptation strategies in semi-arid Pakistan
Source: Sci Rep. 2026 Feb 4;16:4766. doi: 10.1038/s41598-026-36853-z (PMC12873164; doi:10.1038/s41598-026-36853-z)

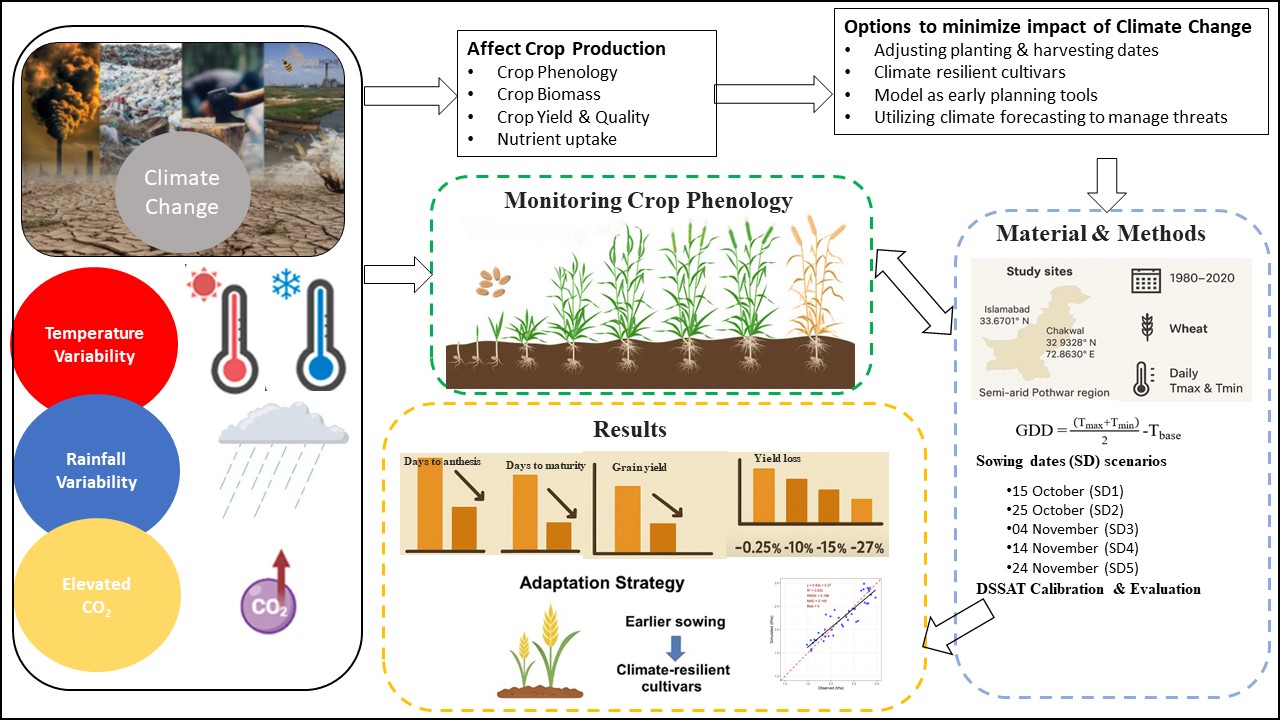

Supplement: Supplementary file 1 — Supplementary Material 1 [file 41598_2026_36853_MOESM1_ESM.jpg]
